# Eugenol Improves Insulin Secretion and Content of Pancreatic Islets from Male Mouse

**DOI:** 10.1155/2020/7416529

**Published:** 2020-08-05

**Authors:** Ali Akbar Oroojan

**Affiliations:** Department of Physiology, Faculty of Medicine, Student Research Committee, Dezful University of Medical Sciences, Dezful, Iran

## Abstract

Glucose homeostasis is required for control of insulin secretion. Phenolic compounds improved glucose-stimulated insulin secretion (GSIS). Eugenol is a phenolic compound that may increase GSIS. So, it was decided to investigate the effect of eugenol on the insulin secretion and content of pancreatic islets from the male mice. In this experimental study, 3-month-old NMRI mice (20–25 g) were prepared. The pancreatic islets of Langerhans were isolated using the collagenase digestion method and divided into 12 groups: glucose 2.8, 5.6, and 16.7 mM, glucose 2.8 mM + eugenol 50, 100, and 200 *µ*M, glucose 5.6 mM + eugenol 50, 100, and 200 *µ*M, and glucose 16.7 mM + eugenol 50, 100, and 200 *µ*M. The islet's insulin secretion and content were measured after 1 hour and 24 hours incubation at 37°C, respectively, by the ELISA assays method and related commercial kit. Present results showed that all doses of eugenol increased islet's insulin secretion and content in the medium containing glucose concentrations 2.8, 5.6, and 16.7 mM (*P *<* *0.05). In conclusion, eugenol as a phenolic compound increased insulin secretion and content of pancreatic islets. The moderate dose of this compound enhanced insulin secretion during hypo- and hyperglycemic conditions, as well as a high dose of eugenol, increased insulin content. Finally, present research suggested that the administration of eugenol 100 *µ*M was suitable for the early stage of T2DM as well as eugenol 200 *µ*M for the advanced stage of this disease.

## 1. Introduction

Glucose homeostasis is required for control of insulin secretion from beta-pancreatic cells. This homeostasis is prepared by glucose, food, and various neurological or hormonal factors [1]. During diabetes mellitus, an increase in blood glucose is occurred due to a lack of insulin secretion or insulin dysfunction or both [2]. In diabetic patients, an increase in insulin secretion compensates for insulin resistance that leads to the decrease of the insulin-secreting cell activity resulting in decreased glucose tolerance. So, this alteration is the primary sign of disease onset. At this time, insulin-secreting cells do not respond well to many drugs such as sulfonylureas [3]. Also, long-term administration of these drugs can induce side effects as well as the reduced performance of these cells [1]. For this reason, several types of research studies have described that the long use of herbal medicines is better in terms of safety and efficacy than chemical drugs to treat various ailments [4]. Eugenol (4-allyl-2-methoxyphenol), as the main constituents of clove *Syzygium aromaticum* (L.), is a phenolic compound belonging to phenylpropanoids. This compound is also found in soy, beans, coffee, cinnamon, basil, bananas, bay leaves, and other foods. The antioxidant activity of these plants has been shown. In addition, the anti-inflammatory activity of *Syzygium aromaticum* is also known, which is associated with the effects of eugenol. Moreover, several other pharmacological activities of eugenol are antitumor, antibacterial, antifungal, antipyretic, anesthetic, and analgesic effects [5].

Because the rodents' pancreatic Langerhans islets have less fibrosis and collagen than humans, they are easier to isolate and are used for research on the effects of hormones secreted by the pancreas [3]. Phenolic compounds have hypoglycemic activities via the inhibition of glucose transport, upregulatory activities of glucose uptake, improved glucose-stimulated insulin secretion (GSIS), or insulin secretion capacity [6]. Therefore, due to the importance of eugenol as a bioactive molecule and its presence in various foods and medicinal plants, the insulin secretion stimulating effects of this compound, and the lack of a study on the direct effect of this substance on insulin secretion from the islets of Langerhans, it was decided to investigate the effect of eugenol on the insulin secretion and content of pancreatic Langerhans islets from the male mouse.

## 2. Materials and Methods

### 2.1. Animals

In this experimental study, 3-month-old NMRI mice (20–25 g) were kept at a 12-hour light-dark cycle and 20°C ± 4°C temperature. The animals were treated in accordance with the principles and guidelines on animal care of Dezful University of Medical Sciences as reviewed by an ethics committee (IR.DUMS.REC.1398.021), as well as free access to tap water and commercial chow ad libitum.

### 2.2. Pancreatic Islets Isolation

After anesthetizing the animals with ketamine (70 mg/kg)/xylazine (10 mg/kg) (Alfasan, Netherlands), the pancreas is removed and placed in a Petri dish containing Krebs-bicarbonate buffer solution (Merck, Germany). The separated pancreas is cut into 1 mm pieces, and the contents of the Petri dish are centrifuged at 100 × g for 5 min. In the next step, the surface of the centrifuged sample is separated, and the remaining contents are transferred to a 15 ml conical tube containing Krebs-bicarbonate buffer plus collagenase (1-2 mg/pancreas) (Roche, Germany) to separate the islets from the exocrine tissue. The incubation time was 15 min at 37°C. Then, 15 mL of cold Krebs-bicarbonate buffer was added to the tube to stop the digestion of collagenase and centrifuged at 500 × g for 5 min. Finally, the islets were transferred to a Petri dish and separated manually using a pipette under a stereomicroscope [7].

### 2.3. Insulin Secretion and Content of Isolated Islets of Langerhans

The pancreatic Langerhans islets are transferred to 2 mL microtubes containing Krebs-bicarbonate buffer in addition to basal 2.8, moderate 6.5, and excitation 16.7 mM concentrations of glucose (Merck, Germany) [8]. Then, 50, 100, and 200 *µ*M of eugenol (Kemdent, United Kingdom) was added to the microtubes and incubated at 37ºC for 60 min [9]. After incubation, the samples were centrifuged at 100 × g for 5 min, and 0.9 mL of supernatant was removed and stored at −70ºC until insulin measurement was performed. A similar protocol is used to evaluate insulin content, except that 0.8 mM hydrochloric acid (HCl) (Merck, Germany) dissolved in ethanol 96% was added to the microtubes after 30 min, and the incubation period was 24 hours. Each microtube contains 7 islets, and the number of samples is repeated 6 times for each group [10].

### 2.4. Grouping of Islets

  Group 1: isolated islets receiving a concentration of 2.8 mM glucose
  Group 2: isolated islets receiving a concentration of 2.8 mM of glucose plus eugenol 50 *µ*M
  Group 3: isolated islets receiving a concentration of 2.8 mM glucose plus eugenol 100 *µ*M
  Group 4: isolated islets receiving a concentration of 2.8 mM glucose plus eugenol 200 *µ*M
  Group 5: isolated islets receiving a concentration of 5.6 mM glucose
  Group 6: isolated islets receiving a concentration of 5.6 mM glucose plus eugenol 50 *µ*M
  Group 7: isolated islets receiving a concentration of 5.6 mM glucose plus eugenol 100 *µ*M
  Group 8: isolated islets receiving a concentration of 5.6 mM glucose plus eugenol 200 *µ*M
  Group 9: isolated islets receiving a concentration of 16.7 mM glucose
  Group 10: isolated islets receiving a concentration of 16.7 mM glucose plus eugenol 50 *µ*m
  Group 11: isolated islets receiving a concentration of 16.7 mM glucose plus eugenol 100 *µ*m
  Group 12: isolated islets receiving a concentration of 16.7 mM glucose plus eugenol 200 *µ*m

### 2.5. Measurement of Islet's Insulin Secreted and Content

The insulin secreted and content of islet were evaluated using the ELISA assays method and a related commercial kit (Monobind, USA) (the sensitivity of hormone detection per assay tube was 0.182 *µ*IU/ml).

### 2.6. Statistical Analysis

Data were statistically analyzed using SPSS software (version 16) with one-way analysis of variance (ANOVA), followed by post hoc least significant difference (LSD) tests. All results were represented as mean ± standard error (SE), and differences were considered statistically significant at *P* < 0.05.

## 3. Results

### 3.1. Effects of Eugenol on Islet's Insulin Secretion

**Figure 1:**
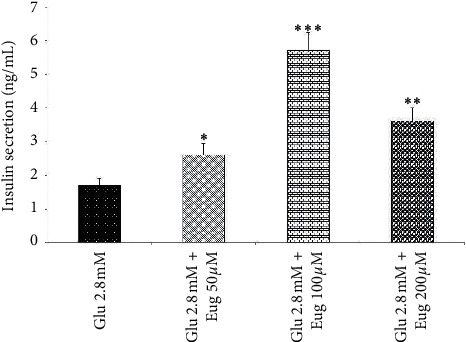
Effects of eugenol on islet's insulin secretion in medium containing glucose 2.8 mM. Data are expressed as the mean ± SEM of 6 samples for islet's insulin secretion (7 islets in each sample). ^*∗*^*P* < 0.05, ^*∗∗*^*P* < 0.01, and ^*∗∗∗*^*P* < 0.001 are significantly different from the glucose 2.8 mM group. Glu: glucose and Eug: eugenol.

**Figure 2:**
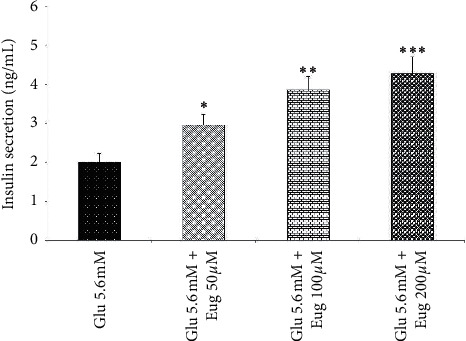
Effects of eugenol on islet's insulin secretion in medium containing glucose 5.6 mM. Data are expressed as the mean ± SEM of 6 samples for islet's insulin secretion (7 islets in each sample). ^*∗*^*P* < 0.05, ^*∗∗*^*P* < 0.01, and ^*∗∗∗*^*P* < 0.001 are significantly different from the glucose 5.6 mM group. Glu: glucose and Eug: eugenol.

**Figure 3:**
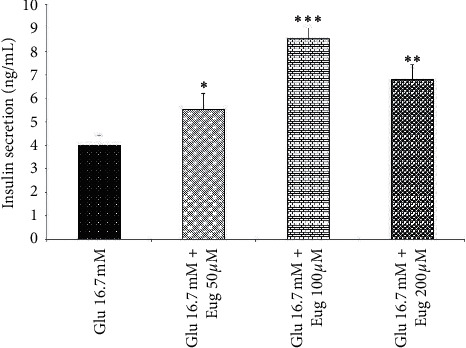
Effects of eugenol on islet's insulin secretion in medium containing glucose 16.7 mM. Data are expressed as the mean ± SEM of 6 samples for islet's insulin secretion (7 islets in each sample). ^*∗*^*P* < 0.05, ^*∗∗*^*P* < 0.01, and ^*∗∗∗*^*P* < 0.001 are significantly different from the glucose 16.7 mM group. Glu: glucose and Eug: eugenol.

Present results showed that eugenol 50, 100, and 200 *µ*M increased islet's insulin secretion in the medium containing glucose concentrations 2.8 and 16.7 mM (*P* < 0.05, *P* < 0.001, and *P* < 0.01, respectively; Figures 1 and 2). The same effect was observed in the medium containing glucose concentration 5.6 mM after eugenol 50, 100, and 200 *µ*M administrations (*P* < 0.05, *P* < 0.01, and *P* < 0.001, respectively; Figure 3).

### 3.2. Effects of Eugenol on Islet's Insulin Content

**Figure 4:**
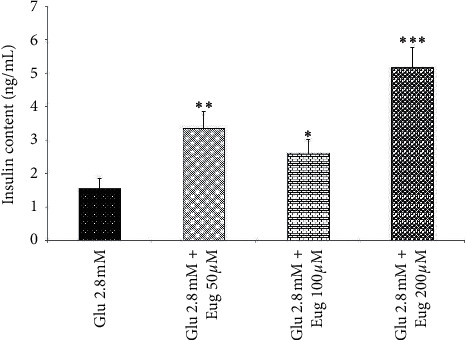
Effects of eugenol on islet's insulin content in medium containing glucose 2.8 mM. Data are expressed as the mean ± SEM of 6 samples for islet's insulin secretion (7 islets in each sample). ^*∗*^*P* < 0.05, ^*∗∗*^*P* < 0.01, and ^*∗∗∗*^*P* < 0.001 are significantly different from the glucose 2.8 mM group. Glu: glucose and Eug: eugenol.

**Figure 5:**
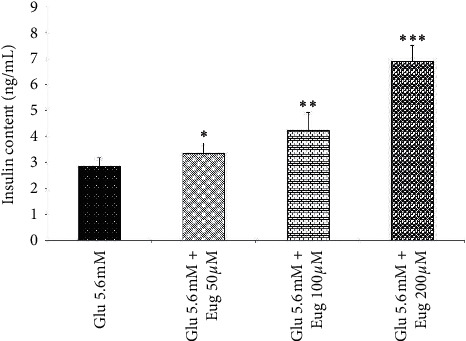
Effects of eugenol on islet's insulin content in medium containing glucose 5.6 mM. Data are expressed as the mean ± SEM of 6 samples for islet's insulin secretion (7 islets in each sample). ^*∗*^*P* < 0.05, ^*∗∗*^*P* < 0.01, and ^*∗∗∗*^*P* < 0.001 are significantly different from the glucose 5.6 mM group. Glu: glucose and Eug: eugenol.

**Figure 6:**
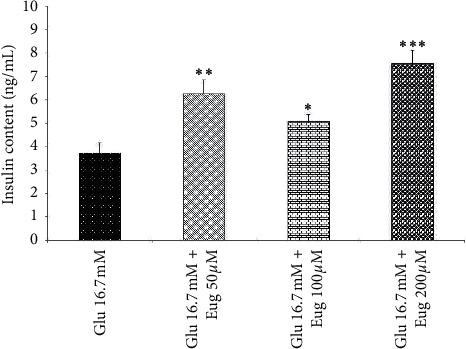
Effects of eugenol on islet's insulin content in medium containing glucose 16.7 mM. Data are expressed as the mean ± SEM of 6 samples for islet's insulin secretion (7 islets in each sample). ^*∗*^*P* < 0.05, ^*∗∗*^*P* < 0.01, and ^*∗∗∗*^*P* < 0.001 are significantly different from the glucose 16.7 mM group. Glu: glucose and Eug: eugenol.

Administration of eugenol 50, 100, and 200 *µ*M increased islet's insulin content in the 2.8 and 16.7 mM medium of glucose concentration (*P* < 0.01, *P* < 0.05, and *P* < 0.001, respectively; Figures 4 and 5). Also, a similar result appeared in the groups that received glucose at a concentration of 5.6 mM along with eugenol 50, 100, and 200 µM (*P* < 0.05, *P* < 0.01, and *P* < 0.001, respectively; Figure 6).

## 4. Discussion

The results of this study indicated that eugenol increased insulin secretion and content from isolated islets of Langerhans. Previous studies showed that eugenol has the antidiabetic effect in diabetic mice that exhibit this effect through inhibition of pancreatic alpha-amylase and the lipase enzyme activity [11]. Also, present data revealed a new antidiabetic function of eugenol, which was to increase the insulin secretion and content of the pancreatic islets. Based on the present study, many phenolic compounds such as epicatechin and quercetin increase GSIS from normal and oxidant-stressed *β*-cell or enhance regeneration of pancreatic *β*-cells and insulin release in diabetic rats [6, 12].

Flavonoids can modulate the release of insulin by changes in Ca^2+^ fluxes through L-type Ca^2+^ channels. It was demonstrated that quercetin and rutin increase insulin secretion from *β*-cells via the intracellular influx of Ca^2+^ by endoplasmic reticulum activating L-type Ca^2+^ channels. Moreover, kaempferol could increase ATP generation in *β*-cells and produce a transcriptional activation of insulin mediated by cyclic adenosine monophosphate (cAMP) signaling, and these processes lead to Ca^2+^ entering inside the cell, activate protein kinase C (PKC) isozymes, and exocytosis of insulin granule [6, 13, 14]. So, since eugenol is a phenolic compound, it can be suggested that a similar mechanism has been occurred to increase the insulin secretion and content of the isolated islet in the present study, but future research studies are required to clarify the exact mechanism of this event.

Pancreatic *β*-cells are susceptible to oxidative stress and free radicals due to their low activity of antioxidant defenses. The oxidative stress condition occurs during exposure of *β*-cells to low and high glucose concentrations that lead to low nicotinamide adenine dinucleotide phosphate (NADPH) or high advanced glycation end products (AGEs) production. Ultimately, these alterations could lead to the disruption of the GSIS in the isolated islets of Langerhans [15]. On the other hand, exogenous antioxidants such as phenolic compounds in different doses and physiologic conditions may have different effects. For example, low doses of carotenoids and polyphenols play an important role in many antioxidant mechanisms in living organisms, but high doses of these compounds may be toxic and act as a prooxidant [16]. So, the results of the present study indicated that eugenol had the greatest effect on GSIS in hypo- and hyperglycemic conditions at a dose of 100 *µ*M and led to the most increase in insulin secretion from the isolated Langerhans islets. Also, this effect may be occurred based on the dose-dependent function of phenolic compounds in hypo- and hyperglycemic conditions.

The release of insulin is pulsatile and rhythmic in nature [17]. The presence of glucose in isolated pancreatic islets medium showed a biphasic pattern of insulin release. The first phase develops rapidly by a burst of insulin secretion, and the second phase occurs followed by a sustained component. Pancreatic *β*-cells contain 2 pools of insulin secretory granules including a reserve pool and a readily releasable pool (RRP). It was revealed that RRP granules are released at the first phase of GSIS, and preparing a subsequent supply for new granules to contain insulin was performed in the second phase [18]. The second phase of pulsatile insulin secretion is related to the islet insulin content [19]. Hence, the insulin content in the medium of isolated islet is consisting of insulin secretion and amount or formation of insulin [20, 21]. Therefore, present results demonstrated that low dose and high dose of eugenol administration were more potent on insulin content of pancreatic islets in hypo- and hyperglycemic conditions, and it can be suggested that these doses of eugenol have more impact on the second phase of insulin release via mobilizing of subsequent supply for producing new granules that contain insulin.

Insulin pulsatility is disrupted during diabetes disease. Decrease of the first phase and low second phase insulin release is characteristic of type 2 diabetes mellitus (T2DM). Also, a decrease in the first phase of GSIS is found in the initial stage of T2DM and prediabetes condition that represents *β*-cells dysfunction, and the second phase of insulin release is destroyed following the progression of diabetes [22, 23]. Finally, this study showed that eugenol administration increased islet's insulin secretion and content due to positive effects on 2 phases of pulsatile insulin release. So, it can be suggested that eugenol 100 *µ*M was more potent for GSIS and early stage of T2DM, as well as eugenol 200 *µ*M for progression or the latest stage of this disease.

## 5. Conclusions

In conclusion, eugenol as a phenolic compound increased insulin secretion and content of pancreatic islets. The moderate dose of this compound enhanced insulin secretion during hypo- and hyperglycemic conditions, as well as high dose of eugenol, increased insulin content. However, the insulin-releasing effect of eugenol was dose-dependent in the normoglycemic condition. Ultimately, present research suggested that the administration of eugenol 100 *µ*M was suitable for the early stage of T2DM as well as eugenol 200 *µ*M for the advanced stage of this disease.

## Data Availability

The data used to support the findings of this study are available from the corresponding author upon request.

## References

[1] Henquin J.-C., Dufrane D., Nenquin M. (2006). Nutrient control of insulin secretion in isolated normal human islets. *Diabetes*.

[2] Gavin J. R., Alberti K., Davidson M. B., DeFronzo R. A. (2000). Report of the expert committee on the diagnosis and classification of diabetes mellitus. *Diabetes Care*.

[3] Katzung B. G. (2001). *Basic and Clinical Pharmacology*.

[4] Dhandapani S., Subramanian V. R., Rajagopal S., Namasivayam N. (2002). Hypolipidemic effect of cuminum cyminum L. on alloxan-induced diabetic rats. *Pharmacological Research*.

[5] Barboza J. N., da Silva Maia Bezerra Filho C., Silva R. O., Medeiros J. V. R., de Sousa D. P. (2018). An overview on the anti-inflammatory potential and antioxidant profile of eugenol. *Oxidative Medicine and Cellular Longevity*.

[6] Soares J. M. D., Leal A. E. B. P., Silva J. C., Almeida J. R., de Oliveira H. P. (2017). Influence of flavonoids on mechanism of modulation of insulin secretion. *Pharmacognosy Magazine*.

[7] O’Dowd J. F. (2009). The isolation and purification of rodent pancreatic islets of Langerhans. *Type 2 Diabetes*.

[8] Ahangarpour A., Oroojan A. A., Badavi M. (2018). Exendin-4 protects mice from D-galactose-induced hepatic and pancreatic dysfunction. *Pathobiology of Aging & Age-Related Diseases*.

[9] Jo H. K., Kim G. W., Jeong K. J., Kim D. Y., Chung S. H. (2014). Eugenol ameliorates hepatic steatosis and fibrosis by down-regulating SREBP1 gene expression via AMPK-mTOR-p70S6K signaling pathway. *Biological and Pharmaceutical Bulletin*.

[10] Machado de Oliveira C. A., Ferreira Paiva M., Alencar Soares Mota C. (2010). Exercise at anaerobic threshold intensity and insulin secretion by isolated pancreatic islets of rats. *Islets*.

[11] Mnafgui K., Kaanich F., Derbali A. (2013). Inhibition of key enzymes related to diabetes and hypertension by Eugenol in vitro and in alloxan-induced diabetic rats. *Archives of Physiology and Biochemistry*.

[12] Yang K., Chan C. B. (2018). Epicatechin potentiation of glucose-stimulated insulin secretion in INS-1 cells is not dependent on its antioxidant activity. *Acta Pharmacologica Sinica*.

[13] Rajagopal S., Fields B. L., Kamatchi G. L. (2014). Contribution of protein kinase C*α* in the stimulation of insulin by the down-regulation of Cav*β* subunits. *Endocrine*.

[14] Kumar D. P., Rajagopal S., Mahavadi S. (2012). Activation of transmembrane bile acid receptor TGR5 stimulates insulin secretion in pancreatic *β* cells. *Biochemical and Biophysical Research Communications*.

[15] Roma L. P., Jonas J.-C. (2020). Nutrient metabolism, subcellular redox state, and oxidative stress in pancreatic islets and *β*-cells. *Journal of Molecular Biology*.

[16] Bouayed J., Bohn T. (2010). Exogenous antioxidants-double-edged swords in cellular redox state: Health beneficial effects at physiologic doses versus deleterious effects at high doses. *Oxidative Medicine and Cellular Longevity*.

[17] Molina P. E., Molina P. E. (2006). *Endocrine Physiology*.

[18] Seino S., Shibasaki T., Minami K. (2011). Dynamics of insulin secretion and the clinical implications for obesity and diabetes. *Journal of Clinical Investigation*.

[19] Song S. H., Rhodes C. J., Veldhuis J. D., Butler P. C. (2003). Diazoxide attenuates glucose-induced defects in first-phase insulin release and pulsatile insulin secretion in human islets. *Endocrinology*.

[20] Blixt M., Niklasson B., Sandler S. (2009). Suppression of bank vole pancreatic islet function by proinflammatory cytokines. *Molecular and Cellular Endocrinology*.

[21] Johannesen J., Helqvist S., Nerup J. (1990). Not all insulin secretagogues sensitize pancreatic islets to recombinant human interleukin 1*β*. *Acta Endocrinologica*.

[22] Satin L. S., Butler P. C., Ha J., Sherman A. S. (2015). Pulsatile insulin secretion, impaired glucose tolerance and type 2 diabetes. *Molecular Aspects of Medicine*.

[23] Pørksen N. (2002). The in vivo regulation of pulsatile insulin secretion. *Diabetologia*.

